# Determining the Effects of Emotional Freedom Techniques on Sexual Dysfunction and Self‐Care Management in Women Diagnosed With Multiple Sclerosis

**DOI:** 10.1002/brb3.70635

**Published:** 2025-06-17

**Authors:** Ayşe Çuvadar, Ayfer Güneş, Yeter Çuvadar Baş, Sezgin Kehaya

**Affiliations:** ^1^ Faculty of Health Sciences, Department of Midwifery Karabük University Karabük Türkiye; ^2^ Department of Neurology Trakya University Medical Research and Application Centre Edirne Türkiye; ^3^ Vocational School Gedik University İstanbul Türkiye

**Keywords:** EFT, multiple sclerosis, self‐care, sexual dysfunction

## Abstract

**Aim:**

This study aims to determine the effects of Emotional Freedom Techniques (EFT) on sexual dysfunction and self‐care in women diagnosed with multiple sclerosis (MS).

**Method:**

This study was conducted using a pretest–posttest experimental design, including follow‐up assessments to evaluate the sustainability of the intervention effects. The sample consisted of 16 women aged between 19 and 49 years who were diagnosed with MS and presented to the neurology clinic of a university hospital in Türkiye between October 2023 and September 2024. Data collection instruments included the Personal Information Form (PIF), Multiple Sclerosis Intimacy and Sexuality Questionnaire‐19 (MSISQ‐19), and Self‐Care Management Process in Chronic Illness (SCMP‐G). Repeated measures analysis of variance (ANOVA) was employed to analyze changes in sexuality and self‐care over time. Statistical significance was set at *p* < 0.05.

**Results:**

Participants received two EFT sessions per month, each lasting 60 min, together with affirmation sessions conducted at least twice a week for 10–15 min. The mean scores of all subdimensions of the MSISQ‐19 reached the lowest levels by the seventh week following EFT intervention, with significant differences between time points (*p* < 0.05). Even though an improvement was observed in the social protection dimension of self‐care, the mean scores for the self‐protection subdimension decreased.

**Conclusion:**

The application of EFT demonstrated positive effects on sexual functioning and self‐care levels in women with MS. Therefore, EFT can be integrated into the holistic care processes of patients with MS.

## Graphical abstract

This study evaluated the effectiveness of Emotional Freedom Techniques (EFT) on sexual dysfunction and self‐care management in women with multiple sclerosis. A total of 16 participants received bi‐monthly EFT sessions and self‐affirmation practices. Results showed significant improvements in sexual function and social self‐care, supporting EFT as an integrative therapeutic method for MS management.

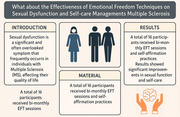

## 1 Introduction

Multiple sclerosis (MS), which affects nearly 2.8 million people throughout the world, is an inflammatory, autoimmune, chronic, and progressive disease that damages the myelin sheaths and axons of the central nervous system, leading to cognitive and functional decline, disability, and a deterioration in the quality of life (Demirci et al. 2024; Gürkan and Özdelikara 2024). MS is known to be three times more prevalent in women than in men, and individuals with MS must effectively adapt to the chronic challenges imposed by the disease (Pourhaji et al. 2024; Rakhshani et al. 2024). Sexual dysfunction is a significant and subtle symptom that is commonly expected in individuals with MS. It was noted that sexual dysfunction contributes to reduced libido, difficulty achieving orgasm, and other sensory disturbances, ultimately leading to a lower quality of life, as well as adverse health, personal relationship, and safety outcomes. The prevalence of sexual dysfunction in women with MS is estimated to range between 55% and 61% (Dourou et al. 2024; Zaloum et al. 2024). Even though the exact etiology of MS is still unknown, psychological factors and stress are thought to influence disease progression and play a significant role in exacerbations and the emergence of new attacks (Rakhshani et al. 2024). Self‐care describes an individual's ability to understand the conditions and factors affecting their health, make decisions aimed at improving treatment, physical and psychosocial outcomes, and lifestyle modifications, and implement these decisions accordingly (Heidari‐Soureshjani et al. 2023; Rakhshani et al. 2024). It is well documented that individuals with chronic diseases such as MS exhibit poorer health behaviors compared to the general population, which, in turn, reduces their self‐care capacity. These patients require both personal and structured treatment plans that enhance their ability to engage in self‐care and slow disease progression, and it is strongly recommended that such treatment plans incorporate relaxation and coordination exercises targeting physical, sensory, motor, and cognitive functions (Dehghani et al. 2023). Due to the progressive nature of MS and the side effects of pharmacological treatments, it has been observed that pharmacological interventions alone may be insufficient in controlling the disease. Experts recommend MS patients adopt complementary therapeutic approaches in addition to medication, including strategies aiming to avoid anxiety, stress, and mental tension (Pourhaji et al. 2024; Rakhshani et al. 2024).

Emotional Freedom Techniques (EFT) is recognized as an evidence‐based psychoeducational intervention that integrates cognitive and exposure‐based approaches with acupressure (Church et al. 2022; Farsi et al. 2024). This method represents a simplified version of Thought Field Therapy (TFT), originally developed by Roger Callahan in the 1980s (Callahan 1985). While the technique involves rhythmic tapping on specific acupressure points using the fingertips, its historical roots can be traced back to traditional practices such as Chinese medicine, Japanese massage, qigong, and yoga (Diamond 1985). The defining characteristic of EFT is this unique application method, widely referred to as “tapping” (Church et al. 2022; Craig 2008/2010).

EFT combines psychological strategies—such as increasing awareness, imaginal exposure, cognitive reframing, preframing, and systematic desensitization—with acupressure performed by tapping specific energy points with the fingertips. This noninvasive tactile stimulation has been shown to enhance therapeutic outcomes (Altuntaş and Düzgüner 2020; Church et al. 2022). While increasingly popular as a self‐help tool used by millions globally, EFT is also implemented in clinical settings and supported by a growing body of empirical evidence demonstrating its efficacy (Feinstein 2021; Gaudiano et al. 2012). Although EFT's core techniques are relatively easy to learn, its effective therapeutic application requires structured and comprehensive training (Craig 2008/2010). Various professional organizations offer certification and training programs; however, there is currently no central authority that universally defines EFT (Feinstein 2009). In 2013, a consensus document officially recognized EFT as an “evidence‐based and manualized approach” (Church et al. 2022).

In clinical practice, EFT is implemented according to standardized protocols validated through scientific research, and these protocols include specific fidelity checks designed to ensure proper therapeutic application by practitioners (Craig 2008/2010). Such procedural structures are regarded as key contributors to the enhanced clinical efficacy of EFT (Church et al. 2022). Previous studies indicated that EFT is effective in reducing fatigue symptoms in women with MS and is considered a safe and appropriate nonpharmacological strategy for self‐management of fatigue and that EFT can be utilized by midwives and nurses as a bedside intervention (Ghaderi et al. 2021; Mehdipour et al. 2022). Furthermore, EFT is employed as a clinical intervention in nursing and midwifery care, education, and research, and is recognized as an effective technique for nurses and midwives to help improve various physical and emotional conditions in their patients (Ghaderi et al. 2021).

The present study was designed to determine the effects of EFT on sexual dysfunction and self‐care in women diagnosed with MS.

## 2 Methods

It is an experimental, pretest–posttest study carried out between October 2023 and September 2024 in the neurology clinic of a university hospital in Türkiye.

### 2.1 Participants

**FIGURE 1:**
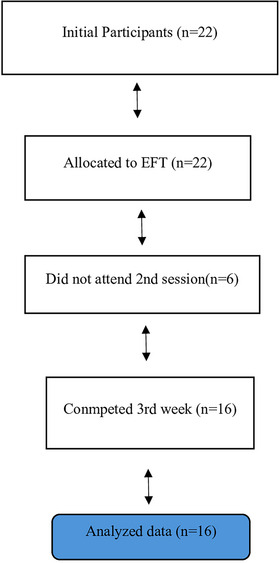
Workflow diagram.

This study employed a pretest–posttest experimental design with follow‐up measurements. The study population consisted of 16 women aged between 19 and 49 years who were diagnosed with MS who presented to the neurology clinic of a university hospital in Türkiye between October 2023 and September 2024. The required sample size was calculated using G^*^Power 3.1.9.7 (Faul et al. 2007). The calculation was based on a large effect size (0.40), a one‐way repeated measures analysis of variance (ANOVA) (within factors), a 5% margin of error (*α* = 0.05), an intermeasurement correlation of 0.5, and 90% power (1‐β = 0.90), yielding a required sample size of 15 (Cohen 1988) (Figure 1). Data collection was conducted via face‐to‐face interviews with women who met the inclusion criteria. The inclusion criteria were set as being over 18 years of age, having a confirmed diagnosis of MS, an Expanded Disability Status Scale (EDSS) score below 6, being female, being sexually active, and voluntarily consenting to participate. The exclusion criteria were set as having a diagnosed psychiatric disorder and experiencing an acute MS attack at the time of the study. Analysis of the participants’ demographic characteristics revealed that the mean age was 37.31 ± 7.95 years (range: 19–49), and the mean age at MS onset was 27.25 ± 5.37 years. Among the participants, 75.0% (*n* = 12) were married, and 37.5% (*n* = 6) had a college‐level or higher education. Regarding financial status, 68.8% (*n* = 11) reported their income as equal to their expenses. In addition, 68.8% (*n* = 11) were nonsmokers, and 87.5% (*n* = 14) reported no alcohol consumption.

### 2.2 Ethical Considerations

The present study was carried out in line with the Declaration of Helsinki on Human Rights. Ethical approval was obtained from the Non‐Interventional Scientific Research Ethics Committee of Trakya University Faculty of Medicine (TÜTF‐GOBAEK 2023/237, Date: October 9, 2023). Prior to participation, written informed consent was obtained from all participants.

### 2.3 Measures

Data collection instruments included the Personal Information Form (PIF), Multiple Sclerosis Intimacy and Sexuality Questionnaire‐19 (MSISQ‐19), and Self‐Care Management Process in Chronic Illness (SCMP‐G) (Doğan 2019; Hançerlioğlu and Aykar 2018). Permissions for the use of the MSISQ‐19 and SCMP‐G were obtained from the respective authors. Before participation, all participants were required to sign an informed consent form. The collected data were recorded in an SPSS database for statistical analysis.

*PIF*: This form, developed by the researchers, includes questions related to the participants’ sociodemographic characteristics.

*MSISQ‐19*: Developed by Foley et al. (2000) to address the impact of MS symptoms on intimate relationships, this scale was adapted into Turkish and validated by Doğan (2019). The MSISQ‐19 consists of 19 items that evaluate primary, secondary, and tertiary sexual dysfunction:

Primary Sexual Dysfunction: Direct loss of sexual function due to the neurological effects of MS. Items: 12, 16, 17, 18, 19 (Total score: 5–25).

Secondary Sexual Dysfunction: Indirect effects of MS symptoms (e.g., fatigue, spasms, bladder/bowel dysfunction). Items: 1, 2, 3, 4, 5, 6, 8, 10, 11 (Total score: 9–45).

Tertiary Sexual Dysfunction: Psychosocial effects of MS (e.g., depression, loss of self‐confidence, fear of rejection). Items: 7, 9, 13, 14, 15 (Total score: 5–25).

The scoring process is conducted using a 5‐point Likert scale (1 = *never*, 5 = *always*), with higher scores indicating a higher level of dysfunction. Symptoms scoring 3 or higher may require further evaluation. The estimated time for completion of the scale is 5–8 min.

*SCMP‐G*: Originally developed by Huffman (1987) as the “Development of an Instrument to Measure Use of Self‐Care Management Processes‐Guarding (SCMP‐G),” this scale was adapted for Turkish populations and validated by Hançerlioğlu and Aykar in 2018. It consists of two subdimensions: self‐protection and social protection, with a total of 35 items. The items are responded to using a 5‐point Likert scale (1 = *strongly disagree*, 5 = *strongly agree*), where higher scores indicate better self‐care management. Even though Cronbach's alpha reliability coefficient for the original scale was 0.85, it was found to be 0.63 in this study.

### 2.4 Procedure

As part of the preintervention phase, participating women completed the PIF, MSISQ‐19, and SCMP‐G.

### 2.5 Intervention Steps

**FIGURE 2:**
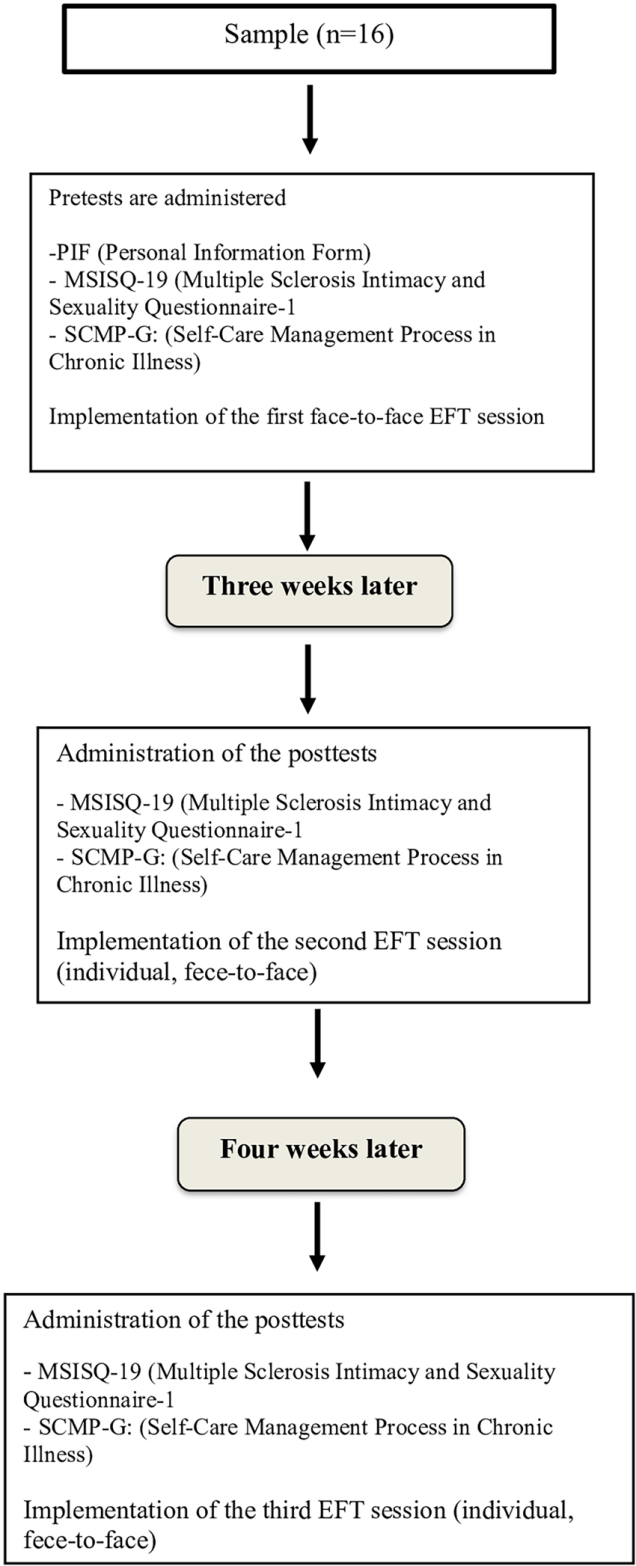
EFT diagram.

Step 1: Baseline assessments were conducted, followed by the first EFT session (face‐to‐face). During the session, tapping was performed on acupressure points located on the head, hands, and torso. Each tap was accompanied by the repetition of a setup statement specifically tailored to the participant (“I am in control of managing my sexual health and self‐care.” “I am improving and feeling stronger every day.” “My body is working to bring me health and peace.” “I approach myself and my body with compassion, and my sexual health is improving every day.”).
Step 2: Three weeks after the first EFT session, the first postintervention assessments were conducted, followed by the second EFT session.
Step 3: Four weeks after the second EFT session, the second postintervention assessments were conducted. During this period, participants were encouraged to use their personalized setup statements as needed (Figure 2).

Each session lasted approximately 45–50 min, and the duration and setup statements were adjusted based on the participant's individual concerns, perspectives, support systems, past traumas, and emotional blockages. The EFT intervention was administered by a certified EFT practitioner, who was also an expert midwife. Sessions were conducted in a one‐on‐one setting within the neurology clinic of the hospital, specifically in the patient's private room.

Homework EFT:

To facilitate self‐practice at home, participants received telephone support twice a week, with each call lasting 5–10 min. During these calls, participants were guided to practice EFT independently using predetermined setup statements.

Key points include:

Crown of the Head: Located at the center of the uppermost part of the head.
Eyebrow Beginning: Positioned just above and slightly to the side of the nose, at the beginning of the eyebrow.
Side of the Eye: Found on the bone at the outer corner of the eye.
Under the Eye: Situated on the bone directly below the eye.
Under the Nose: Located between the nose and the upper lip.
Chin: Positioned between the lower lip and the bottom of the chin.
Collarbone: At the junction of the sternum, collarbone, and first rib.
Under the Arm: Approximately four finger‐widths beneath the armpit, along the side of the torso.
Fingers: When applying tapping, individuals should use the sides of their fingers, excluding the ring finger. This technique involves gently tapping each point five times using the fingertips, which are generally applied in a specific sequence. In addition, the karate point, located on the edge of the palm, just below the little finger, is also used in this process.

When tapping on these points, individuals should use the tips of two or more fingers, tapping each location about five times. While some points, such as the beginning of the eyebrow, the side of the eye, and under the eye, have corresponding points on the opposite side of the body, tapping on one side is sufficient. However, if both hands are free, it is possible to tap both sides simultaneously.

### 2.6 Data Analysis

The data analysis process was conducted using IBM SPSS Statistics 27.0 (IBM Corp., Armonk, NY). Descriptive statistics were presented as mean ± standard deviation (SD) or percentages (%). The normality of data distribution was examined using the Shapiro–Wilk test (*p* > 0.05). Repeated measures ANOVA was conducted to analyze changes in sexual function and self‐care over time. Statistical significance was set at *p* < 0.05.

## 3 Results

Analysis of the participants’ demographic characteristics revealed that the mean age was 37.31 ± 7.95 years (min: 19; max: 49), and the mean age at MS onset was 27.25 ± 5.37 years. Of the participants, 75.0% (*n* = 12) were married, and 37.5% (*n* = 6) had a college‐level or higher education.

Regarding financial status, 68.8% (*n* = 11) reported their income was equal to their expenses, while 68.8% (*n* = 11) were nonsmokers, and 87.5% (*n* = 14) did not consume alcohol.

Considering the distribution by MS type, 37.5% (*n* = 6) had relapsing‐remitting MS, 31.3% (*n* = 5) had secondary progressive MS, and 31.3% (*n* = 5) had primary progressive MS. Regarding treatment methods, 18.8% (*n* = 3) were on oral therapy, 62.4% (*n* = 10) received intravenous treatment, and 18.8% (*n* = 3) were on subcutaneous therapy (Table 1).

**TABLE 1:** Personal characteristics of women (*n* = 16).

Variable	Mean ± SD
Age	37.31 ± 7.95
MS age	27.25 ± 5.37
*n* (%)	
Marital status Married Single	12 (75.0) 6 (25.0)
Educational level Middle school High school College and higher	5 (31.3) 5 (31.3) 6 (37.5)
Family type Nuclear family Extended family	12 (75.0) 4 (25.0)
Income status Income = expenses Income > expenses Income < expenses	11 (68.8) 2 (12.5) 3 (18.7)
Smoking Yes No	5 (31.2) 11 (68.8)
Alcohol consumption Yes No	2 (12.5) 14 (87.5)
MS type Relapsing‐remitting Secondary progressive Primary progressive	6 (37.5) 5 (31.3) 5 (31.3)
MS treatment Oral Intravenous Subcutaneous	3 (18.8) 10 (62.4) 3 (18.8)

**FIGURE 3:**
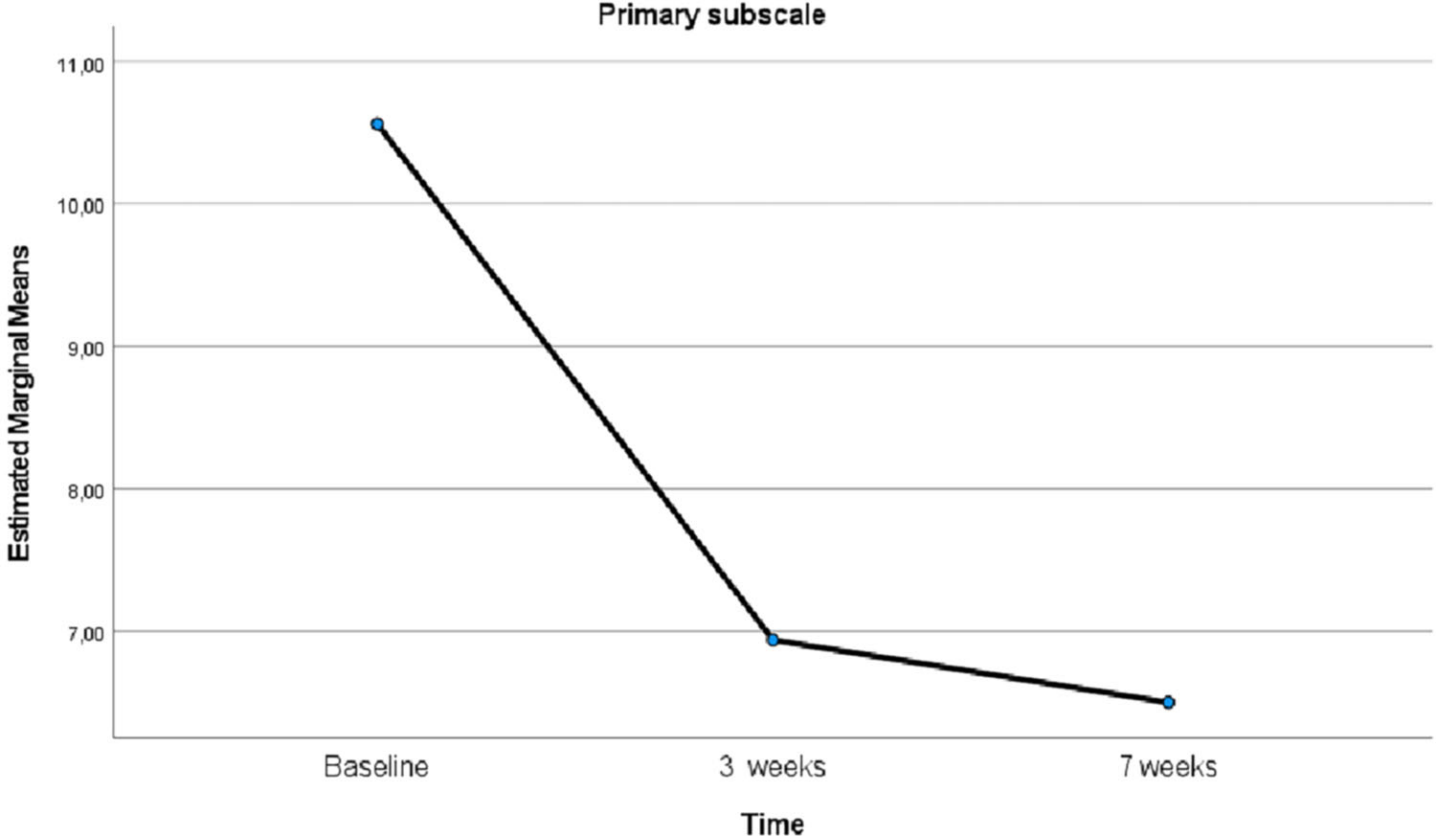
Comparison of the mean scores of the primary subscale of MSISQ‐19 relative to baseline over time.

The changes in the primary subscale levels of MSISQ‐19 before, at Week 3, and at Week 7 following EFT implementation were examined using one‐way repeated measures ANOVA. Examination of the box plot graphs revealed no outliers. The Mauchly's test of sphericity revealed a violation of the sphericity assumption (*χ* ^2^ = 40.791, *p* < 0.001). Consequently, the Greenhouse‐Geisser correction was performed, and the epsilon (*ε*) value was calculated to be 0.701. After applying the correction, the analysis demonstrated a significant difference in sexual function levels over time (*F* = 18.492, *p* < 0.001, *η* ^2^ = 0.552). Subsequent pairwise comparisons employing the Bonferroni correction yielded the following results: The mean primary subscale score at Week 7 after EFT was significantly lower than the scores at baseline and at Week 3 after EFT (*p* = 0.001, *p* = 0.011). In addition, the mean primary subscale score at Week 3 after EFT was significantly lower than the baseline score (*p* = 0.004) (Table 2). The line graph showing the changes over time in the mean scores of the primary subscale of the MSISQ‐19 is presented in Figure 3.

**TABLE 2:** MSISQ‐19 subscale scores after two EFT sessions (mean + SD) (*n* = 16).

MSISQ‐19	Baseline	3 weeks	7 weeks	*p*
Primary subscale	10.56 ± 4.11	6.93 ± 1.80	6.50 ± 1.63	< 0.001
Secondary subscale	17.62 ± 6.82	15.18 ± 5.81	12.56 ± 3.14	< 0.001
Tertiary subscale	10.68 ± 3.38	8.43 ± 2.18	6.25 ± 1.48	< 0.001

**FIGURE 4:**
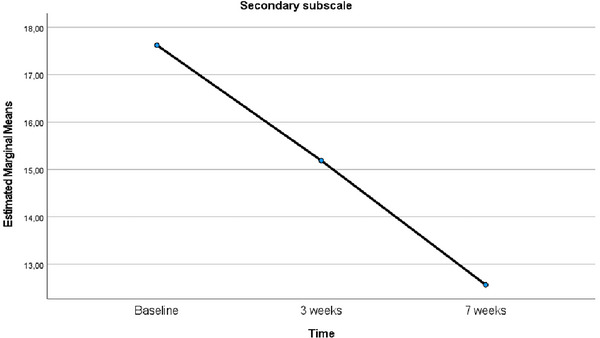
Comparison of the mean scores of the secondary subscale of MSISQ‐19 relative to baseline over time.

Given the results of the repeated measurements variance analysis on changes in the secondary subscale levels of MSISQ‐19 before, at Week 3, and at Week 7 following EFT implementation, Mauchly's test of sphericity confirmed that the sphericity assumption was met (*χ* ^2^ = 4.988, *p* = 0.083). The analysis results indicated a significant difference in sexual function levels over time (*F* = 16.233, *p* < 0.001, *η* ^2^ = 0.520). The mean secondary subscale score at Week 7 after EFT was significantly lower than both the score at Week 3 after EFT and the baseline score (*p* = 0.006, *p* = 0.001) (Table 2). The line graph showing the changes over time in the mean scores of the secondary subscale of the MSISQ‐19 is presented in Figure 4.

**FIGURE 5:**
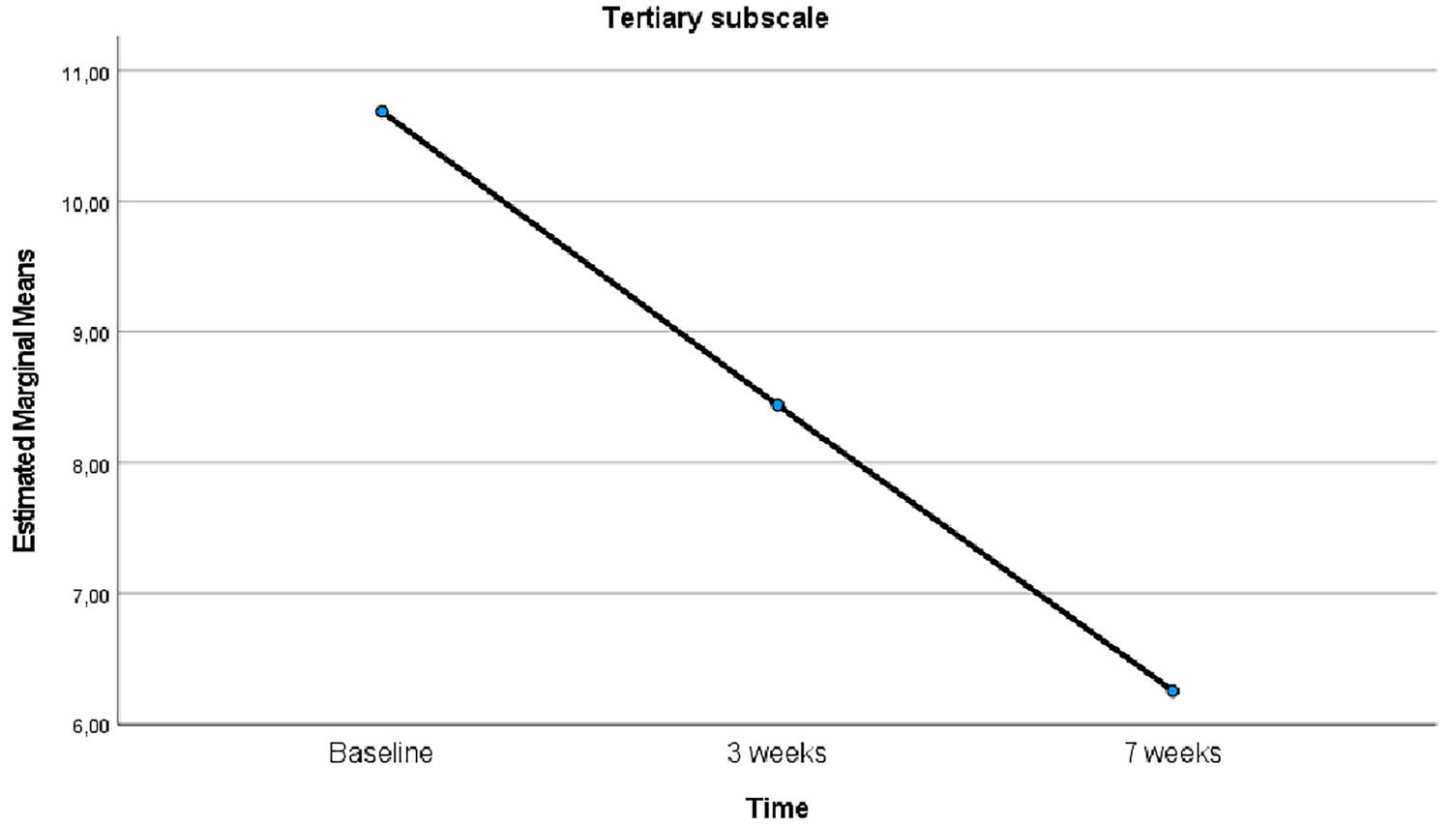
Comparison of the mean scores of the tertiary subscale of MSISQ‐19 relative to baseline over time.

Considering the results obtained from the analysis of changes in the tertiary subscale levels of MSISQ‐19 before, at Week 3, and at Week 7 following EFT implementation, Mauchly's test of sphericity revealed a violation of the sphericity assumption (*χ* ^2^ = 7.798, *p* = 0.020). As a result, the Greenhouse‐Geisser correction was applied, and the *ε* value was calculated to be 0.514. Following the correction, the analysis demonstrated a significant difference in sexual function levels over time (*F* = 17.610, *p* < 0.001, *η* ^2^ = 0.540). Pairwise comparisons using the Bonferroni correction yielded that the mean tertiary subscale score at Week 7 post‐EFT was significantly lower than both the score at Week 3 after EFT and the baseline score (*p* < 0.001) (Table 2). The line graph showing the changes over time in the mean scores of the tertiary subscale of the MSISQ‐19 is presented in Figure 5.

**FIGURE 6:**
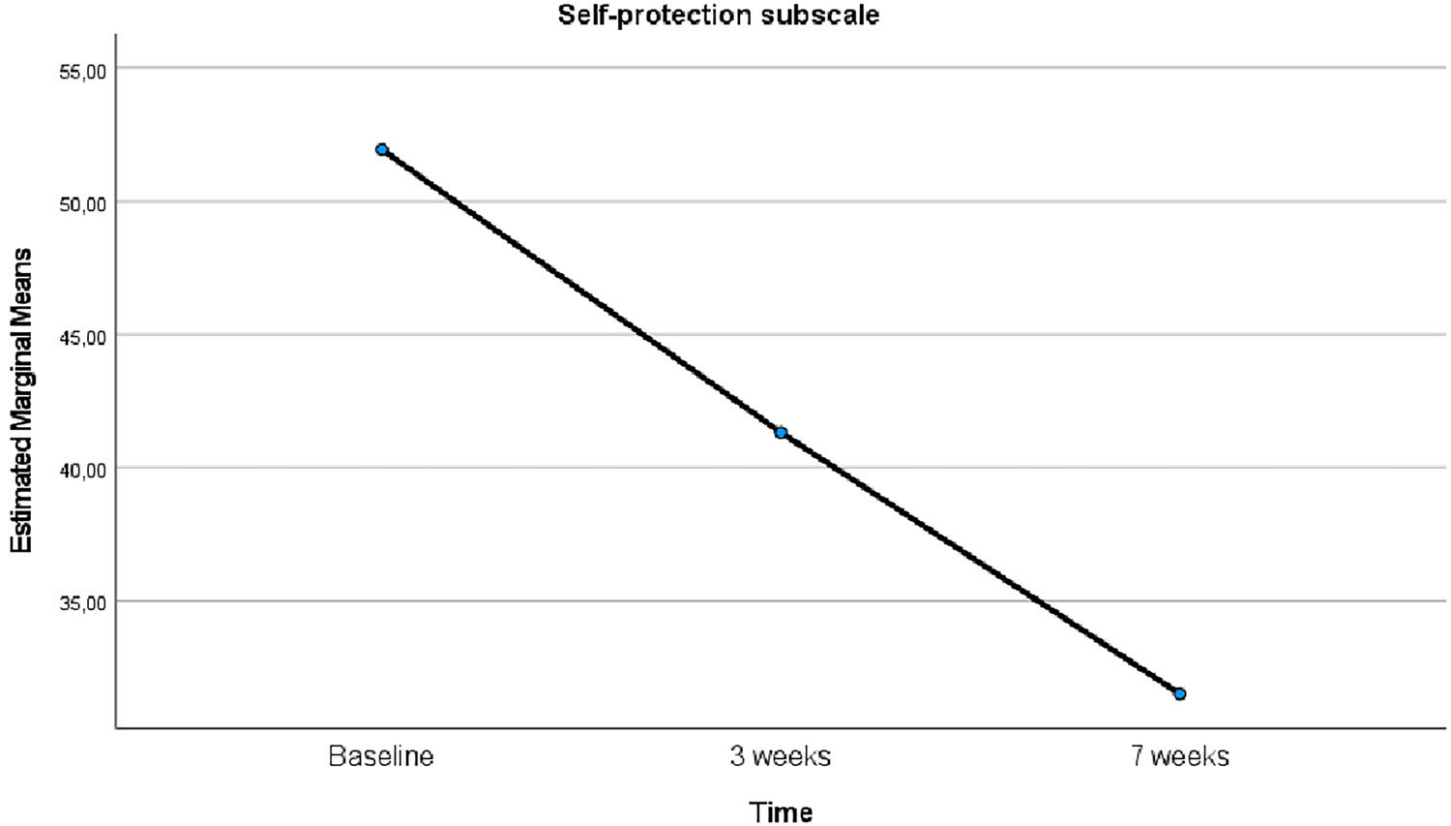
Comparison of the mean scores of the self‐protection subscale of SCMP‐G relative to baseline over time.

The analysis of changes in self‐protection subscale levels before, at Week 3, and at Week 7 following EFT implementation confirmed that the sphericity assumption was met according to Mauchly's test (*χ* ^2^ = 1.585, *p* = 0.453). The analysis results indicated a significant difference in self‐protection levels over time (*F* = 68.384, *p* < 0.001, *η* ^2^ = 0.820). The mean self‐protection subscale score at Week 7 after EFT was significantly lower than both the score at Week 3 after EFT and the baseline score (*p* < 0.001) (Table 3). The graph showing the time‐dependent changes in the mean scores of the self‐protection subscale of the SCMP‐G is presented in Figure 6.

**TABLE 3:** Self‐care management process in chronic illness (SCMP‐G).

SCMP‐G	Baseline	3 weeks	7 weeks	*p*
Self‐protection subscale	51.93 ± 3.23	41.31 ± 11.42	31.50 ± 10.19	< 0.001
Social protection subscale	53.06 ± 4.97	65.25 ± 9.63	74.31 ± 13.01	< 0.001

**FIGURE 7:**
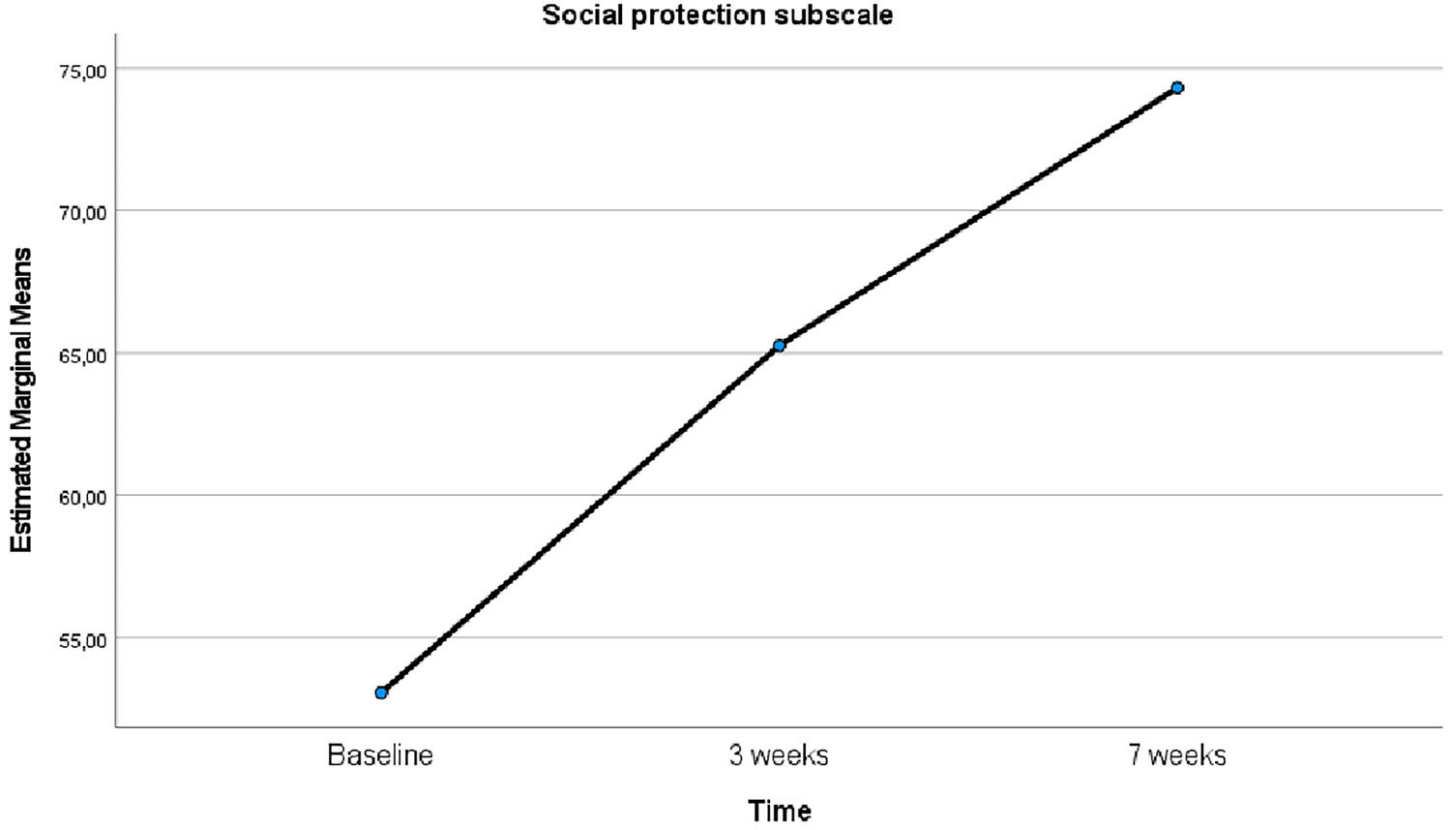
Comparison of the mean scores of the social protection subscale of SCMP‐G relative to baseline over time.

In the analysis conducted to examine the change in subdimension levels of social protection before EFT intervention, at Week 3, and at Week 7, the sphericity test revealed that the assumption of sphericity was not met (*χ* ^2^ = 22.668, *p* < 0.001). Consequently, the Greenhouse‐Geisser correction was applied, and *ε* was found to be 0.555. Following this adjustment, the analysis revealed a significant difference in social protection levels over time (*F* = 45.002, *p* < 0.001, *η* ^2^ = 0.750). Accordingly, pairwise comparisons conducted employing the Bonferroni correction yielded the following results: The mean scores for the social protection subdimension at Week 7 post‐EFT were significantly higher than both the baseline and Week 3 post‐EFT mean scores (*p* < 0.001). Moreover, the mean score at Week 3 post‐EFT was significantly higher than the mean score at Week 7 post‐EFT, with the difference being significant (*p* < 0.001) (Table 3). The graph showing the time‐dependent changes in the mean scores of the social protection subscale of the SCMP‐G is presented in Figure 7.

## 4 Discussion

In this study, which involved implementing two 60‐min EFT protocol sessions per month and affirmation sessions lasting 10–15 min at least twice a week, the mean primary subscale score of the MSISQ‐19 reached its lowest level in the seventh week after EFT, with a significant difference observed across different time points. In the literature, there are many studies addressing sexual dysfunction in women diagnosed with MS (Mohammadi et al. 2020; Nosrat et al. 2020). In a study designed to investigate sexuality‐related factors in women affected by MS, the MSISQ‐19 questionnaire was used to assess its impact on sexual satisfaction and function. Supporting the results achieved in this study, early interventions—particularly mental health support—were highlighted as essential for mitigating the adverse effects of MS on sexuality in women (Mohammadi et al. 2020). Similarly, a study carried out by Nazari et al. (2020) showed that EFT reduces fatigue levels in individuals with MS, indirectly alleviating its impact on sexual function. It was suggested that EFT may serve as a mechanism for regulating energy balance, thereby mitigating neurological symptoms that contribute to sexual dysfunction. Howard (2021) emphasized that interventions promoting body awareness and self‐acceptance in women with MS can enhance sexual health, with changes in body image playing a crucial role in increasing sexual sensitivity.

In this study, the mean secondary subscale score of the MSISQ‐19 was also found to be at its lowest level in the seventh week after EFT. Sezginer (2007) reported that 66.6% of MS patients experience severe difficulties in their sexual lives due to symptoms such as fatigue and muscle stiffness. Energy‐based approaches, such as EFT, were found effective in alleviating these symptoms, which may, in turn, support improvements in sexual function. Another study indicated that self‐management support programs enhance MS patients’ ability to cope with physical challenges, emphasizing that the indirect effects of such programs on sexuality should not be overlooked (Al Abbad 2020). In addition, psychological therapies were shown to alleviate secondary symptoms such as fatigue and muscle stiffness in MS patients, which may contribute to reducing sexual dysfunction (Fragkiadaki et al. 2023). Addressing secondary symptoms, such as fatigue and spasms, can enhance sexual satisfaction in individuals with MS.

In this study, the mean score on the tertiary subscale of the MSISQ‐19 was found to be at its lowest in the 7th week following EFT. Another study examined the impact of MS on couples’ relationships and sexual dysfunction was largely attributed to a lack of communication with one's partner (Esmail 2005). Psychological interventions may help mitigate the effects of tertiary symptoms by addressing communication difficulties. Another study demonstrated that psychological therapies help individuals reconnect with their bodies and alleviate their emotional burdens (Fragkiadaki et al. 2023). There is a clear need for large‐scale studies investigating the effects of EFT on the subscales of the MSISQ‐19.

In this study, the mean score on the self‐protection subscale of the SCMP‐G was at its lowest in the seventh week after EFT. In the study carried out by Nazari et al. (2020), EFT was found to reduce fatigue and depression in individuals with MS, leading to increased energy levels. The lower self‐protection scores may be associated with participants’ perception that they require fewer self‐protection behaviors in their daily lives. The short‐term relief provided by EFT may play a role in this result. Another study revealed that Acceptance and Commitment Therapy (ACT) enhances psychological capital (self‐efficacy, resilience) in individuals with MS and fosters the development of a positive life perspective (Eskandari et al. 2021). Similarly, another study revealed that individuals with MS tend to have low resilience levels, which, in turn, increases their anxiety and depression levels. Psychological interventions were highlighted as crucial tools in mitigating these effects (Ghadbeigi et al. 2024). Furthermore, another study reported that Mindfulness‐Based Cognitive Therapy (MBCT) reduces emotional exhaustion and improves quality of life in individuals with MS (Dizaj Khalili et al. 2022). The indirect effect of EFT on self‐protection may be linked to an enhanced ability to cope with stress, leading individuals to perceive a reduced need for self‐care.

In the present study, the mean score of the social protection subscale of the SCMP‐G was found to be highest at the seventh week after EFT. A previous study demonstrated that ACT enhances psychological capital components such as resilience and self‐efficacy in individuals with MS and contributes to more effective utilization of social support systems (Eskandari et al. 2021). Another study reported that MBCT and visual restructuring therapies reduce emotional exhaustion and lead to positive changes in social relationships among MS patients (Dizaj Khalili et al. 2022). Given these results, it can be thought that EFT facilitates individuals’ ability to benefit from social support systems through similar mechanisms. In a study carried out by Ghadbeigi et al. (2024), the resilience levels of MS patients were found to be positively associated with social support systems, and these support networks significantly reduced levels of depression and anxiety. The high scores observed in the social protection subscale may indicate that EFT helps individuals establish stronger connections with communities and support groups. Furthermore, another study reported that psychological therapies enhance partner and family support in individuals with MS, leading to improvements in personal domains such as sexual function (Fragkiadaki et al. 2023). These results suggest that social support contributes to improvements not only in physiological aspects but also in the psychosocial dimensions of individuals’ quality of life.

## 5 Conclusion

This study assessed the effects of EFT on sexual dysfunction and self‐care management in women diagnosed with MS. The results indicate that EFT leads to significant improvements in primary, secondary, and tertiary sexual dysfunction subdomains and positively influences the social protection subscale of self‐care management. These results suggest that EFT may serve as an effective supportive intervention in enhancing the quality of life of MS patients. However, further studies with larger sample sizes are needed to validate these findings.

### 5.1 Limitations

This study's lack of a randomized controlled design limits the generalizability of the results in terms of causal relationships. Furthermore, the small sample size restricts the statistical power, thereby limiting the applicability of the results to a broader population. Since the data were collected from individuals receiving care at a university hospital, the findings may not be fully generalized to different sociodemographic groups.

Nevertheless, this study holds significant value as the first in the literature to examine the effects of EFT on sexual dysfunction and self‐care in women with MS. In this regard, it provides a foundation for future randomized controlled trials and broader research in this field.

## Author Contributions

**Ayşe Çuvadar**: conceptualization, investigation, writing – original draft, methodology, validation, visualization, writing – review and editing, software, formal analysis, project administration, data curation, supervision. **Ayfer Güneş**: conceptualization, investigation, writing – original draft, methodology, validation, visualization, writing – review and editing, data curation, supervision. **Yeter Çuvadar Baş**: investigation, validation, visualization, writing – review and editing, data curation, supervision. **Sezgin Kehaya**: investigation, visualization, writing – review and editing, data curation, supervision.

## Conflicts of Interest

The authors declare no conflicts of interest.

## Peer Review

The peer review history for this article is available at https://publons.com/publon/10.1002/brb3.70635

## Data Availability

The data that support the findings of this study are available from the corresponding author upon reasonable request.
